# Capturing the free energy of transition state stabilization: insights from the inhibition of mandelate racemase

**DOI:** 10.1098/rstb.2022.0041

**Published:** 2023-02-27

**Authors:** Stephen L. Bearne

**Affiliations:** ^1^ Department of Biochemistry and Molecular Biology, Dalhousie University, Halifax, Nova Scotia, Canada B3H 4R2; ^2^ Department of Chemistry, Dalhousie University, Halifax, Nova Scotia, Canada B3H 4R2

**Keywords:** enzyme inhibition, transition state stabilization, transition state analogue inhibitors, binding free energy, mandelate racemase, inhibitor design

## Abstract

Mandelate racemase (MR) catalyses the Mg^2+^-dependent interconversion of (*R*)- and (*S*)-mandelate. To effect catalysis, MR stabilizes the altered substrate in the transition state (TS) by approximately 26 kcal mol^–1^ (–Δ*G*_tx_), such that the upper limit of the virtual dissociation constant of the enzyme-TS complex is 2 × 10^–19^ M. Designing TS analogue inhibitors that capture a significant amount of Δ*G*_tx_ for binding presents a challenge since there are a limited number of protein binding determinants that interact with the substrate and the structural simplicity of mandelate constrains the number of possible isostructural variations. Indeed, current intermediate/TS analogue inhibitors of MR capture less than or equal to 30% of Δ*G*_tx_ because they fail to fully capitalize on electrostatic interactions with the metal ion, and the strength and number of all available electrostatic and H-bond interactions with binding determinants present at the TS. Surprisingly, phenylboronic acid (PBA), 2-formyl-PBA, and *para*-chloro-PBA capture 31–38% of Δ*G*_tx_. The boronic acid group interacts with the Mg^2+^ ion and multiple binding determinants that effect TS stabilization. Inhibitors capable of forming multiple interactions can exploit the cooperative interactions that contribute to optimum binding of the TS. Hence, maximizing interactions with multiple binding determinants is integral to effective TS analogue inhibitor design.

This article is part of the theme issue ‘Reactivity and mechanism in chemical and synthetic biology’.

## 1. Introduction: benchmarks for transition state analogue design

**Figure 1.**
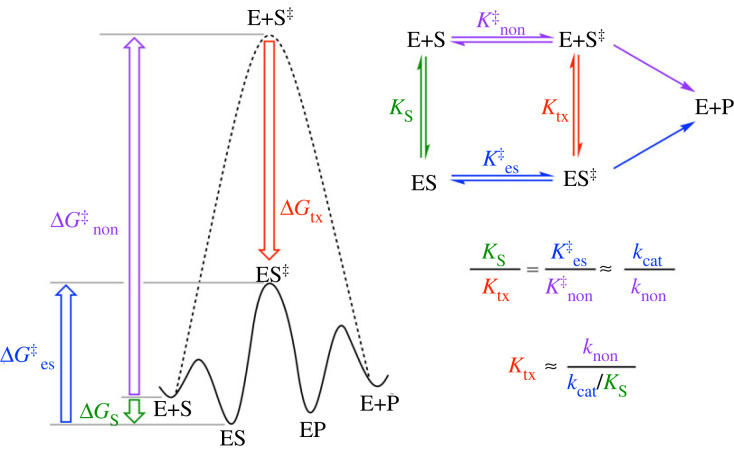
Thermodynamic cycle relating the parameters from TS theory and the thermodynamic parameters for the enzyme-catalysed and the corresponding non-enzymatic (uncatalysed) reactions. *K*_S_ and *K*_tx_ are the dissociation and virtual dissociation constants for the enzyme complexed with substrate (i.e. *K*_m_ ≈ *K*_S_) and the altered substrate in the TS, respectively. *k*_non_ is the rate constant for the uncatalysed reaction and *k*_cat_ is the turnover number [4–6]. (Online version in colour.)

It has been almost three-quarters of a century since Linus Pauling pointed out that ‘enzymes are molecules that are complementary in structure to the activated complexes of the reactions that they catalyze, that is, to the molecular configuration that is intermediate between the reacting substances and the products of reaction for these catalyzed processes' [1,2] [3, p. 709]. Pauling's prescient remarks were placed on a more quantitative footing by Wolfenden [4,5] and Lienhard [6] using transition state (TS) theory and a thermodynamic cycle (figure 1) to relate an enzyme's catalytic efficiency (*k*_cat_/*K*_m_) to its ability to bind the altered substrate in the TS; i.e. *K*_tx_ = *k*_non_/(*k*_cat_/*K*_m_), where *k*_non_ is the rate constant for the corresponding uncatalysed reaction [7]. Recognition that the lower limit for the binding affinity of the altered substrate in the TS, as estimated by the virtual dissociation constant *K*_tx_, was inversely proportional to the rate enhancement (*k*_cat_/*k*_non_) afforded by an enzyme led to the notion that analogues of the TS, or high-energy intermediates of similar structure [8], should be bound tightly by an enzyme [4,9]. Because most biological reactions proceed extremely slowly in the absence of an enzyme [10], remarkably huge rate enhancements on the order of 10^6^- to 10^18^-fold have been reported for enzyme-catalysed reactions such that affinities of 10 nM to 10^–3^ yM are estimated for formation of complexes between enzymes and their altered substrates in the TS [11,12]. This suggests that TS analogue inhibitors could be bound with similar affinities to the extent that a stable analogue of the TS can mimic the geometric and electronic features of the enzymatic TS and effectively capture the available binding energy. Indeed, over the past 50 years, numerous TS analogue inhibitors have been developed targeting enzymes from all five major enzyme classes [13–17], with some providing the basis for the development of drugs [18–23].

**Figure 2.**
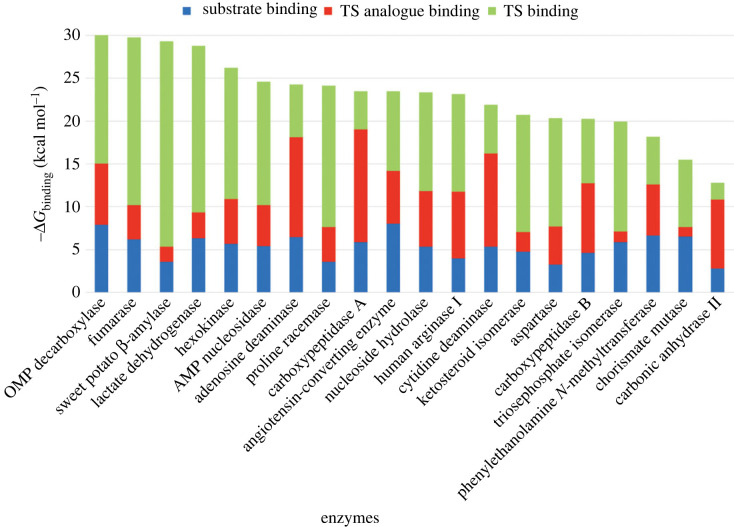
Free energy changes accompanying binding of the substrate (blue), intermediate/TS analogue inhibitor (red) and the altered substrate in the TS (green) for various enzymes. The specific TS analogue inhibitors, as well as references for the corresponding *k*_cat_/*K*_m_, *K*_m_ and *k*_non_ values, are found in the electronic supplementary material. (Online version in colour.)

To establish benchmarks for judging the catalytic power of enzymes, Wolfenden and co-workers measured the non-enzymatic rate constants for a variety of model reactions corresponding to enzyme-catalysed reactions [10,24,25]. These studies revealed that while enzymes have evolved with catalytic efficiencies that typically fall within a narrow range of 2–3 orders of magnitude with the second-order rate constant for encounter of the enzyme and substrate in solution (approx. 10^9^ M^–1^ s^–1^) as an upper limit [26], the rate constants for the corresponding uncatalysed reactions can vary over at least 16 orders of magnitude. Consequently, the variability of the catalytic proficiency (1/*K*_tx_) among enzymes arises primarily owing to differences in their uncatalysed rates [27]. As such, it is the value of *k*_non_ that furnishes the benchmark for judging the catalytic power (i.e. proficiency) of a given enzyme and its potential susceptibility to inhibition by TS analogue inhibitors. With *K*_tx_ values approaching 10^–27^ M for some enzymes [12], the anticipated binding of TS analogue inhibitors could indeed be dramatic relative to the substrate. This raises the question: how much of the available free energy of TS stabilization (Δ*G*_Tx_ = *RT*ln*K*_tx_) do known TS analogue inhibitors capture upon binding their respective enzymes and how does this compare to the change in free energy accompanying substrate binding (Δ*G*_S_)? Figure 2 illustrates the proportion of binding free energy captured upon binding of the most potent intermediate/TS analogue inhibitors with a selection of enzymes for which the *k*_non_ values are known and for which the assay conditions used to determine the catalytic efficiency (*k*_cat_/*K*_m_) and competitive inhibition constant (*K*_i_) are closely matched, if not identical. Not unexpectedly, the free energy changes accompanying substrate binding fall within a limited range of approximately –3 to –8 kcal mol^–1^; however, the free energy changes accompanying binding of the altered substrate in the TS (Δ*G*_tx_) span a wide range from approximately –16 to –39 kcal mol^–1^. For the enzymes listed, the most potent intermediate/TS analogue inhibitors identified capture between 18 and 85% of the available TS stabilization energy (average = 50 (±19)%). On the other hand, the substrates in the ground state capture only 12–42% of the available TS stabilization energy (average = 24 (±7)%). Clearly, there is room for improvement since much of the free energy of TS stabilization remains unused by the intermediate/TS analogue inhibitors presented in figure 2.

To inform the design of TS analogue inhibitors, kinetic isotope effects studies, combined with quantum mechanical modelling, have been employed to estimate the extent of bond breaking and formation at the TSs of enzyme-catalysed reactions [28–30]. Much of this work has been conducted by Schramm and co-workers and has led to the development of extremely potent TS analogue inhibitors directed primarily at enzymes involved in purine and pyrimidine metabolism [22]. While these studies furnished important insights into the design of TS analogues, the techniques are not easily applied in most laboratories and some enzyme-catalysed reactions are not readily amenable to such an approach. Most often, enzymologists resort to designing potential TS analogue inhibitors using only the surmised structure of the TS or intermediate of an enzyme-catalysed reaction as a guide. Herein, I review work from our laboratory that has focused on developing TS analogue inhibitors of mandelate racemase (MR) to address the question: for a mechanistically ‘simple’ enzyme acting on a small molecule, is there a limit to the extent that the free energy of TS stabilization can be captured by an intermediate/TS analogue to effect strong binding?

## 2. The burden borne by mandelate racemase

**Figure 3.**
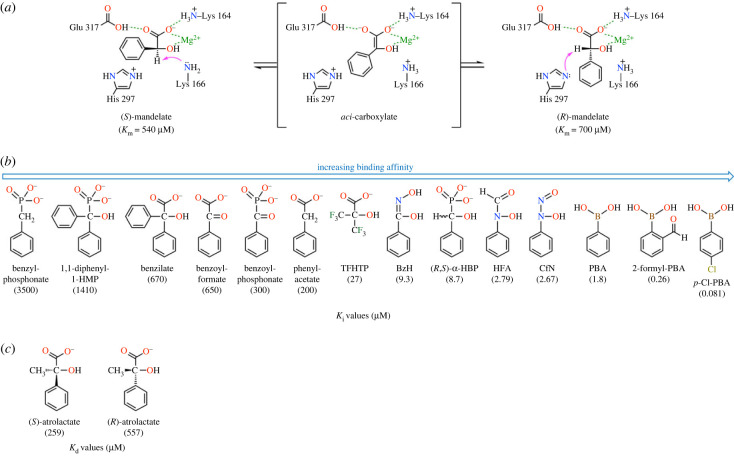
Mechanism for MR-catalysed interconversion of (*S*)- and (*R*)-mandelate (*a*), structures and competitive inhibition constants (*K*_i_, or *K*_i_* for 2-formyl-phenylboronic acid) for various inhibitors of MR (*b*), and *K*_d_ values for the substrate analogues (*S*)- and (*R*)-atrolactate (*c*) [38]. (Online version in colour.)

MR (EC 5.1.2.2) from *Pseudomonas putida* catalyses the Mg^2+^-dependent interconversion of the enantiomers of mandelic acid [31,32]. The enzyme is part of the mandelate pathway that converts the enantiomeric pair of mandelic acids to benzoic acid, which is further catabolised to yield succinate and acetyl-CoA by the enzymes of the β-ketoadipate pathway [31,33,34]. Isotope exchange, site-directed mutagenesis and X-ray crystallographic studies revealed that the enzyme uses a two-base mechanism to catalyse the 1,1-proton transfer reaction [35–37]. At the active site, Lys 166 and His 297 act as enantiospecific Brønsted acid-base catalysts, abstracting the α-proton from (*S*)- and (*R*)-mandelate, respectively, to form an enolic/ate intermediate (figure 3) [39]. The ability of MR to catalyse rapid heterolytic C–H bond cleavage from a carbon acid with a p*K*_a_ value of approximately 29 [40–42] has made MR a useful paradigm for understanding enzyme-catalysed proton abstraction from carbon acids [43–45]. This is especially important considering the abundance of such reactions in enzyme mechanisms and the extremely high p*K*_a_ values typically ranging between 13 and 30 for carbon acid substrates in aqueous solution [46].

In the absence of an enzyme, the rate constant (*k*_non_) for the non-enzymatic racemization of mandelate is 3 × 10^–13^ s^–1^, which corresponds to a free energy of activation equal to 35 kcal mol^–1^ [47]. To effect efficient deprotonation of mandelate, MR must reduce this activation barrier by overcoming two challenges. The most significant challenge is that the enol or enolate intermediate is unstable and poses a huge thermodynamic problem for the enzyme [46,48–50]. Hence, MR must stabilize the intermediate/TS through electrostatic stabilization and H-bonding interactions [50,51], as well as by enhancing the basicity of the Brønsted base catalyst [52,53]. A less significant challenge is that the rate of non-enzymatic abstraction of the α-proton from a carbon acid is usually slower than the rate of abstraction of a proton from a heteroatom or normal acid of equal acidity because of the larger intrinsic barrier for proton abstraction from a carbon acid (approx. 12 kcal mol^–1^) relative to that of a normal acid (≤3 kcal mol^–1^) [54–56]. This poses a kinetic problem for the enzyme.

MR overcomes these challenges to afford a rate enhancement (*k*_cat_/*k*_non_) exceeding 15 orders of magnitude, stabilizing the altered substrate in the TS by approximately 26 kcal mol^–1^ [47,57]. This remarkable proficiency corresponds to an upper limit for the virtual dissociation constant for the enzyme and the altered substrate in the TS (i.e. *K*_tx_) equal to a value of 2 × 10^–19^ M and, therefore, MR is expected to be extremely sensitive to inhibition by analogues of either the TS or the high-energy intermediate formed during catalysis. To design such analogues, knowledge of the electronic and geometric characteristics of the enolic/ate intermediate or TS is required. Gerlt and Gassman proposed that concerted general acid-general base catalysis promotes both the enolization of the mandelate anion and ketonization of the enolic/ate intermediate [42,58–60], while Guthrie & Kluger [51] argued that the principal source of catalysis is electrostatic stabilization. Probably, the interaction of an H-bond donor with the enolate oxygen at an enzyme active site contributes very little to reduction of the intrinsic barrier [61]; while electrostatic interactions that stabilize the π-system of the enolate may make a more significant contribution [62,63]. Substrate and solvent deuterium isotope effect experiments conducted using the MR variants H297N [64], E317Q [65], and K166R [37] further suggest that the reaction is stepwise. Thus, although the precise structure of the TS for the enolization reaction catalysed by MR is not known; for TS analogue design purposes, it is reasonable to expect that the TS resembles either the putative *aci*-carboxylate intermediate (figure 3) or one of its conjugate acids. (For the present discussion, the term ‘*aci*-carboxylate’ is not meant to imply the extent of proton transfer from the general acid catalyst Glu 317 at the active site to the oxygen atom of the intermediate [60].)

## 3. Ground state analogues

To obtain a better understanding of the protein-ligand interactions that contribute to stabilization of the altered substrate in the TS by MR, we explored the ability of carboxylate- and phosphonate-based analogues of the substrate, product and *aci*-carboxylate intermediate to inhibit MR (figure 3) [66]. MR binds phenylacetate (*K*_i_ = 200 µM) and benzoylformate (*K*_i_ = 650 µM) with affinities similar to that observed for the substrate (for MR, *K*_m_ ≈ *K*_S_ [67]). Interestingly, in the ground state, the α-OH group does not appear to make a significant contribution to binding; however, it is required for catalysis since MR does not catalyse exchange of the α-protons of phenylacetate with deuterated solvent [36]. While benzoylformate has an sp^2^ centre at the α-carbon, the observed binding affinity suggests that MR recognizes this ligand as a ground state analogue rather than a structural mimic of the *aci*-carboxylate. Examining the binding of phosphonate analogues was of particular interest since such analogues could serve as structural and electronic mimics of the putative *aci*-carboxylate intermediate. Interestingly, the dianionic benzylphosphonate (*K*_i_ = 3500 µM) was bound more weakly than the substrate, while benzoylphosphonate exhibited a binding affinity (*K*_i_ = 300 µM) similar to that of the substrate. In accord with the observation that the α-OH group makes little contribution to ground state binding but is required for catalysis, (*R*,*S*)-α-hydroxybenzylphosphonate (α-HBP) was a potent inhibitor (*K*_i_ = 8.7 µM), suggesting that α-HBP was recognized by MR as an intermediate/TS analogue (*vide infra*) [66].

In addition to the observations above indicating that the α-OH plays a significant role in stabilizing the altered substrate in the TS, elimination of the interaction between Asn 197 and the α-OH by substitution of Ala in place of Asn indicated that the interaction furnishes approximately 3.5 kcal mol^–1^ of TS stabilization free energy [66]. As such, we explored how variations of substituents at the α-carbon might affect binding by conducting a ‘fluorinated phosphonate scan’ [68] using a series of mono- and di-substituted α-fluorobenzylphosphonates (α-FBP) since fluorine is often employed as an isosteric replacement for OH groups [69]. (*R*,*S*)-α-FBP (*K*_i_ = 810 µM), (*R*)-α-FBP (*K*_i_ = 1110 µM), and (*S*)-α-FBP (*K*_i_ = 530 µM), as well as α,α-diflurobenzylphosphonate (α,α-F_2_BP, *K*_i_ = 1.2 × 10^4^ µM) were all competitive inhibitors of MR, but were bound with affinities that were similar to or much weaker than the binding affinity of mandelate [70]. In part, this lower binding affinity arises from the slight reduction of the p*K*_a_ of the phosphonate group as a result of fluorine substitution at the α-position [71,72]. Our observation that the binding affinities of α,α-F_2_BP and (*S*)-α-FBP increased 12- and 6-fold, respectively, upon reducing the pH of the assay solution from 7.5 to 6.3, indicating that MR exhibits a preference for the phosphonate monoanion. Interestingly, MR exhibited a 2-fold binding preference for (*S*)-α-FBP over (*R*)-α-FBP, unmasking a functional asymmetry at the active site of this pseudosymmetric enzyme (*vide infra*). These results are consistent with the loss of coordination of the Mg^2+^ ion owing to the absence of the α-OH group, as well as possible loss of interaction with Asn 197 since the α-fluoro group can only act as an H-bond acceptor. Despite its C_α_–H bond being more polarized [73] than in mandelate, (*R*)-α-FBP was not a substrate for MR since no change in the ellipticity of (*R*)-α-FBP was observed upon incubating the compound with the enzyme.

## 4. Substrate-product analogues

The hydrophobic pocket that envelopes the phenyl ring of the substrate at the active site of MR is able to accommodate a variety of aryl- and heteroaryl-substituted mandelate derivatives, which serve as substrates to varying degrees [39,57,74–78]. Most surprisingly, the hydrophobic pocket was able to accommodate the two phenyl rings of benzilate (*K*_i_ = 670 µM, figure 3) [79]. This striking observation suggested that as the Walden inversion was effected by MR, the phenyl ring could potentially move from an *R*-pocket to an *S*-pocket within the active site and *vice versa*, with the phenyl rings of benzilate simultaneously occupying the *R*- and *S*-pockets to inhibit the enzyme. Subsequent site-directed mutagenesis experiments, wherein steric bulk was introduced into the putative *S*-pocket formed, in part, by the 50s residues (i.e. the F52W, Y54W and F52W/Y54W variants), revealed that the slightly higher affinity of the wild-type enzyme for (*S*)-mandelate (Km(S)−man=540 μM)  over (*R*)-mandelate (Km(R)−man=700 μM)  could be reversed to yield preferential binding of (*R*)-mandelate by the variants [79]. Interestingly, steric obstruction of the putative *R*-pocket, which is comprised of the 20s residues from the active-site flap, did not produce a pronounced corresponding preference for binding of (*S*)-mandelate probably owing to flap mobility compensating for the increased size of the hydrophobic side chains [80]. Our attempt to enhance binding by substituting the carboxylate of benzilate by a phosphonate group (i.e. 1,1-diphenyl-1-hydroxymethylphosphonate (1,1-diphenyl-1-HMP), *K*_i_ = 1410 µM) to mimic a portion of the structure of the *aci*-caboxylate intermediate did not yield enhanced binding as it did for α-HBP (*vide infra*), suggesting that simultaneous binding of the two phenyl groups obviates mimicry of the *aci*-carboxylate group by the phosphonate group [81].

**Figure 4.**
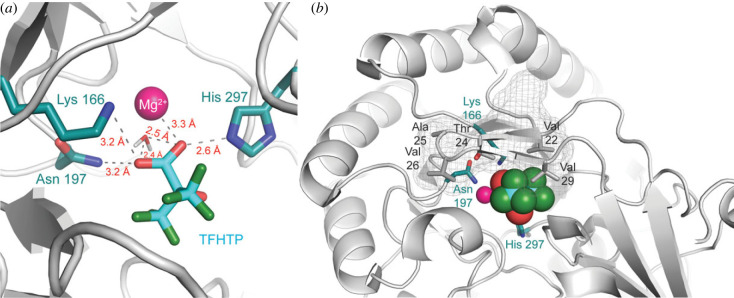
MR with bound TFHTP (Protein Data Bank (PDB) ID: 4FP1 [83]). Panel (*a*) shows the substrate–product analogue bound with its carboxylate bridging the Brønsted acid-base catalysts Lys 166 and His 297. The carboxyl group does not directly chelate the Mg^2+^ ion owing to the intervening water molecule. In panel (*b*), TFHTP (space-filling representation) is shown bound at the mouth of the α/β-barrel. The two trifluoromethyl groups are packed against the active site flap (20s loop, cartoon and mesh representations) and interacting with the hydrophobic side chains of Val 22, 26 and 29, and the methyl groups of Thr 24 and Ala 25 through dispersion forces. In both panels, the Mg^2+^ ion is represented as a magenta sphere. (Online version in colour.)

During our investigation of the ability of the hydrophobic pocket of MR to accommodate various hydrophobic groups on the substrate, we discovered that β,γ-unsaturation was not an absolute requirement for catalysis by MR (cf. ref. [78]) and that MR was capable of racemizing the enantiomers of trifluorolactate (TFL) [82]. Although the *k*_cat_ values for this substrate in both reaction directions were reduced approximately 100-fold relative to those for mandelate, the *K*_m_ values of 1700 µM and 1200 µM for (*S*)- and (*R*)-TFL, respectively, suggested that the active-site hydrophobic pocket bound the trifluoromethyl group as well as the phenyl ring of mandelate. Considering the inhibitory effect of benzilate, we rationalized that an inhibitor of MR might be generated by replacing the two phenyl rings of benzilate with trifluoromethyl groups. Indeed, the so-called substrate-product analogue of TFL, i.e. 3,3,3-trifluoro-2-hydroxy-(trifluoromethyl)propanoate (TFHTP), was a potent inhibitor of MR (*K*_i_ = 27 µM) [83], on par with the intermediate/TS analogue inhibitors (*vide infra*)! The X-ray crystal structure of the MR·TFHTP complex solved to 1.68-Å resolution revealed that TFHTP assumed a novel binding mode at the active site with the two trifluoromethyl groups intimately packing against the 20s loop and with the carboxylate group forming a salt bridge between the two Brønsted acid-base catalysts Lys 166 and His 297 (figure 4). Consequently, the carboxylate and α-OH groups do not chelate the Mg^2+^ ion as observed in all previous structures with bound substrate [37], substrate analogues [36,65,84,85], and intermediate/TS analogues [86]. Hence, the high binding affinity exhibited by TFHTP arises primarily from dispersion forces between the two trifluoromethyl groups and the hydrophobic side chains emanating from residues of the active-site flap.

## 5. Intermediate/transition state analogues: structural and electronic mimicry of the *aci*-carboxylate intermediate

### (a) α-Hydroxybenzylphosphonate

Based on the electronic and geometric character of the enol(ate) intermediate (i.e. the *aci*-carboxylate), one can design putative intermediate/TS analogue inhibitors [8]; however, because the substrate, *aci*-carboxylate, and product have very similar structures, the number of structural variations available is quite limited since the analogue must be approximately isosteric with the intermediate. A phosphonate group was employed to mimic the dianionic character of the *aci*-carboxylate (figure 3). (*R*,*S*)-α-HBP was a potent competitive inhibitor of MR with a *K*_i_ value of 4.7 µM and 3.9 µM when assayed in the *R*→*S* and *S*→*R* reaction directions, respectively, using the circular dichroism-based assay (or 8.7 µM when assayed in the *R*→*S* reaction direction using a high performance liquid chromatography-based assay) [66]. Inhibition assays conducted with the partially resolved (*R*)-α-HBP (76% ee) and (*S*)-α-HBP (82% ee) gave *K*_i_ values of approximately 34 µM and 1.1 µM, respectively, in the *R*→*S* reaction direction, indicating that (*S*)-α-HBP was the more potent inhibitor. Such a strong binding preference for one enantiomer over the other is unexpected for a ‘pseudosymmetric’ enzyme such as MR (i.e. approximately equal *k*_cat_ and *K*_m_ values in both the *R*→*S* and *S*→*R* reaction directions) [32], although such enantioselective binding preferences were also noted for the competitive inhibitors (*R*)- and (*S*)-atrolactate, as well as the irreversible inhibitor (*R*)-phenylglycidate [36].

Interestingly, the pH dependence of the inhibition of MR by (*R*,*S*)-α-HBP revealed that more potent inhibition arose when the phosphonate was in its monoanionic form [66] as was also observed for the α-fluoro-substituted phosphonate analogues discussed above. This result was surprising since the phosphonate dianion was anticipated to more closely resemble the electronic character of the intermediate. However, unlike the carboxylate or *aci*-carboxylate groups in which the negative charge is rotationally symmetrical about the line which bisects the angle made by the carboxyl carbon and the two anionic oxygens, the phosphonate monoanion is not rotationally symmetric. Consequently, orientation of the monoanionic phosphonate function so that the vector of its negative charge aligns with that of the substrate or *aci*-carboxylate intermediate results in skewed binding of this analogue. Indeed, such an altered binding orientation may account for the enantioselective binding of the enantiomers of α-HBP and why the (*R*,*S*)-α-HBP monoanion is an inhibitor of MR and not a substrate as evidenced by the lack of MR-catalysed exchange of the α-H with solvent deuterium in buffered D_2_O [66].

### (b) Benzohydroxamate

Recognizing the importance of the α-OH for binding and the need to mimic the planar structure of the *aci*-craboxylate, benzohydroxamate (BzH) was examined as an inhibitor wherein the hydroxamate function was substituted for the glycolate moiety of the substrate (figure 3). Like α-HBP, BzH was bound approximately 100-fold more tightly than the substrate with a *K*_i_ value of 9.3 µM [66]. The pH dependence of the inhibition revealed that MR preferentially bound the deprotonated form of BzH. Beyond the greater binding of BzH relative to mandelate, additional evidence supporting the notion that MR recognizes BzH as a structural and electronic mimic of the TS was apparent from the linear free energy relationship [87,88] between the efficiency of variant MRs and their corresponding binding affinities with BzH. The values of log(*k*_cat_/*K*_m_) for wild-type MR and 20 variants varied linearly with the corresponding values of log(1/*K*_i_) with a slope of 1.01 ± 0.14 (*r*^2^ = 0.74), while the linear dependence of values of log(1/*K*_m_) with the corresponding values of log(1/*K*_i_) only had a slope of 0.25 ± 0.14 with much weaker correlation (*r*^2^ = 0.14) [86]. Hence, BzH is not a ground state analogue but exhibits mimicry of the altered substrate in the TS.

**Figure 5.**
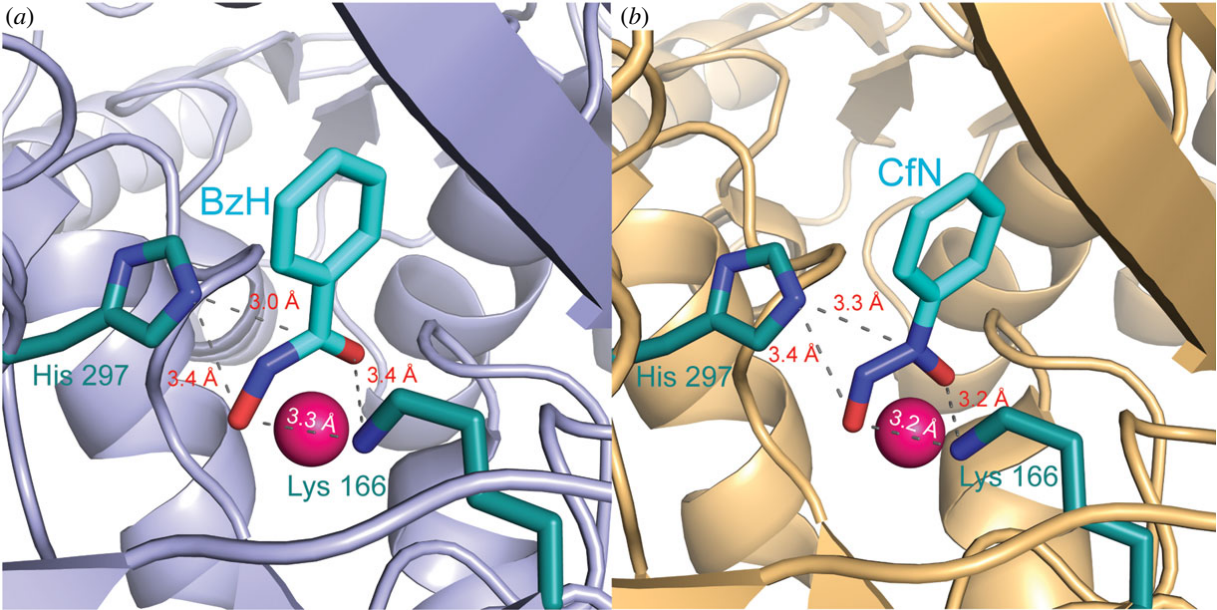
MR with bound intermediate/TS analogues BzH and CfN. Both BzH (panel (*a*), PDB ID: 3UXK [86]) and CfN (panel (*b*), PDB ID: 3UXL [86]) chelate the Mg^2+^ ion and are bound in a conformation with the phenyl ring coplanar with the hydroxamate and diazeniumdiolate moieties, respectively, thereby mimicking the expected conformation of the *aci*-carboxylate intermediate. (Online version in colour.)

The X-ray crystal structure of the MR·BzH complex was determined to 2.20-Å resolution (Protein Data Bank (PDB) ID: 3UXK, figure 5*a*) [86]. As expected for the intermediate, BzH was bound in a planar conformation with the hydroxamate moiety chelating the Mg^2+^ ion at the active site. Enhanced interaction of the *aci*-carboxylate with the Mg^2+^ ion is an expected feature of TS stabilization since both the intermediate and TS bear additional negative charge relative to mandelate in the ground state. Indeed, the distances between the Mg^2+^ and the chelating oxygen atoms of the carboxylate and α-OH groups of bound substrate analogue (*S*)-atrolactate are 2.2 and 2.3 Å, respectively, while the corresponding distances in the MR·BzH complex are 2.2 and 2.1 Å (PDB ID: 1MDR [36]), respectively, suggesting that the interactions of BzH with the Mg^2+^ are slightly stronger than those in the ground state. The N*^ζ^* and N*^ε^*^2^ atoms of Lys 166 and His 297, respectively, were positioned equidistant from the α-carbon of BzH, consistent with the expectation that, for a pseudosymmertric enzyme, these two acid-base catalysts would be equally poised to protonate the α-carbon of the *aci*-carboxylate intermediate. Interestingly, the size of the hydrophobic cavity was observed to contract from approximately 39 Å^3^ in the MR·(*S*)-atrolactate ground state complex [36] to approximately 29 Å^3^ in the MR·BzH complex. Although much of the reduction in volume arose from movement of the Lys 166 side chain, there was a notable reduction in the volume of the hydrophobic cavity surrounding the phenyl moiety consistent with dispersion forces contributing to TS stabilization [77].

### (c) Cupferron

Cupferron (CfN), which bears the diazeniumdiolate (nitroso-hydroxylamine) group [89], also mimics the electronic and structural character of the *aci*-carboxylate intermediate and competitively inhibits MR with a *K*_i_ value of 2.67 µM (figure 3) [90]. The p*K*_a_ of CfN is 4.16 [91], hence the inhibitor exists as a monoanion over the pH range for which MR is active. The X-ray crystal structure of MR complexed with CfN was determined to 2.20-Å resolution (PDB ID: 3UXL) and revealed that this intermediate analogue, like BzH, was bound in a planar conformation with the diazeniumdiolate moiety chelating the Mg^2+^ ion in a bidentate fashion (figure 5*b*) [86]. Relative to the distances between the Mg^2+^ and the chelating oxygen atoms of the carboxylate (2.2 Å) and α-OH (2.3 Å) groups of bound (*S*)-atrolactate, the corresponding distances in the MR·CfN complex were reduced to 2.0 Å and 2.1 Å, respectively. Thus, similar to BzH, the interaction of CfN with the Mg^2+^ is slightly stronger than that observed between Mg^2+^ and the ground state analogue (*S*)-atrolactate. Also similar to the observations made with the MR·BzH complex, the MR·CfN complex exhibited nearly equal proximity of the N*^ζ^* and N*^ε^*^2^ atoms of Lys 166 and His 297, respectively, to the nitrogen atom attached to the phenyl ring of CfN, as well as contraction of the volume of the hydrophobic pocket from approximately 39 Å^3^ in the MR·(*S*)-atrolactate complex [36] to approximately 25 Å^3^ in the MR·CfN complex [86].

### (d) *N*-Hydroxyformanilide

Reverse or retro-hydroxamates are potent inhibitors of a variety of enzymes, including metalloenzymes [92]. *N*-Hydroxyformanilide (HFA) was a competitive inhibitor of MR (*K*_i_ = 2.79 µM), binding with an affinity similar to BzH and CfN (figure 3). Crystal structures of metalloenzymes with bound reverse hydroxamate inhibitors reveal that the reverse hydroxamate moiety often coordinates the metal ion in a bidentate manner with the *N*-hydroxyl group in its deprotonated form [93]. When the pH dependence of the inhibition of MR by HFA was examined, it was found that MR bound both the protonated form of HFA (*K*_i_ = 9 µM) and its conjugate base (*K*_i_ = 0.91 µM), with a 10-fold binding preference for the latter form [90]. Although an X-ray crystal structure of MR with bound HFA is not currently available, it is likely that the reverse hydroxamate function coordinates the Mg^2+^ ion as its *Z*-(*syn*) conformational rotomer in a manner similar to BzH and CfN despite the *E*-(*anti*) rotomer being favoured in aqueous media [94,95].

### (e) Why do these intermediate/transition state analogues fall short of the mark?

Overall, as analogues of the *aci*-carboxylate intermediate, BzH, CfN and HFA all bind MR with similar *K*_i_ values, ranging between 2 and 10 µM. The *Z*-forms of BzH, CfN and HFA mimic the structure and planarity of the *aci*-carboxylate intermediate and permit bidentate coordination of the Mg^2+^ ion, but, as monoanions, they fail to mimic the negative charge that is delocalized over the dianionic *aci*-carboxylate intermediate. The additional oxygen and negative charge present in the *aci*-carboxylate intermediate, but absent in these inhibitors, would be anticipated to contribute substantially to the free energy of TS binding through simultaneous interactions with the Mg^2+^ ion and the adjacent electrophilic catalyst Glu 317 [51,65]. Consequently, the binding of these inhibitors captures only approximately 30% of the approximately 26 kcal mol^–1^ used to bind the altered substrate in the TS.

## 6. Boronic acids: clasping the catalytic machinery

**Figure 6.**
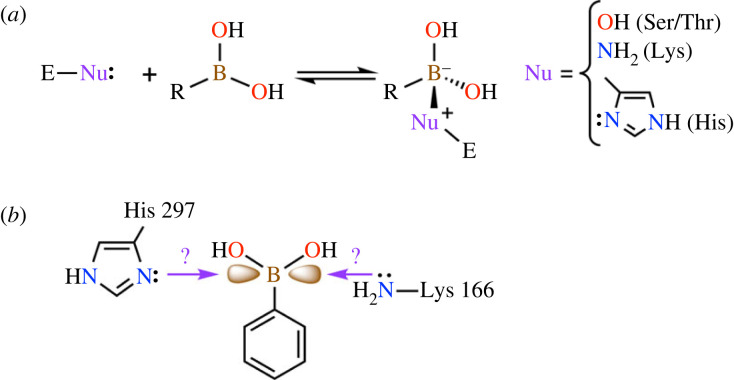
Interaction of hydrolases with boronic acid inhibitors (*a*) and hypothesized interaction of MR with boronic acids (*b*). (Online version in colour.)

The use of boronic acid-based inhibitors as TS analogues targeting hydrolases has proved quite successful [96–98]. Since the boron atom acts as a Lewis acid, its vacant p orbital can readily accept electrons from donor atoms typically located on the side chains of Ser, Thr, His or Lys residues to form a coordinate (dative) bond (i.e. N–B interaction) with concomitant conversion of the boron atom from a neutral sp^2^ centre to an anionic sp^3^ centre (figure 6*a*). The latter species mimics the geometric and electronic features of the tetrahedral intermediate(s) and/or TSs formed during hydrolysis. Moreover, hydroxyl groups covalently linked to the boron atom serve as additional points of molecular recognition by accepting or donating H-bonds. For these reasons, and its low toxicity [98], interest in incorporating the boronic acid functionality into drug molecules has grown over the past decade [99–101].

### (a) Phenylboronic acid

Considering the architecture of the active site of MR, we hypothesized that positioning a boron atom at the location normally occupied by the α-carbon of mandelate would potentially permit N–B interactions [102] with the two active-site Brønsted base catalysts (i.e. Lys 166 and His 297) (figure 6*b*). Gratifyingly, we found that derivatives of phenylboronic acid (PBA) were potent, reversible competitive inhibitors of MR, with binding affinities exceeding the binding affinity of *aci*-carboxylate analogues by approximately 1–2-orders of magnitude (figure 3) [103]. For a series of *para*-substituted PBA derivatives, both larger electron-donating (i.e. OCH_3_ and CH_3_) and electron-withdrawing (i.e. CN, NO_2_, and CF_3_) substituents decreased the binding affinity relative to PBA probably owing to adverse steric effects at the active site, while halogens were well tolerated at the *para*-position with *p*-Cl-PBA exhibiting the most potent inhibition (*K*_i_ = 81 nM). Thus, the binding affinity of *p*-Cl-PBA exceeded that of the substrate by 1.23 × 10^4^-fold. Interestingly, methylboronic acid itself was a very weak inhibitor of MR (*K*_i_ ≈ 0.13 M), indicating that the boronic acid moiety is not solely responsible for the potent inhibition but that the synergistic binding contribution from the phenyl ring is also essential [104].

**Figure 7.**
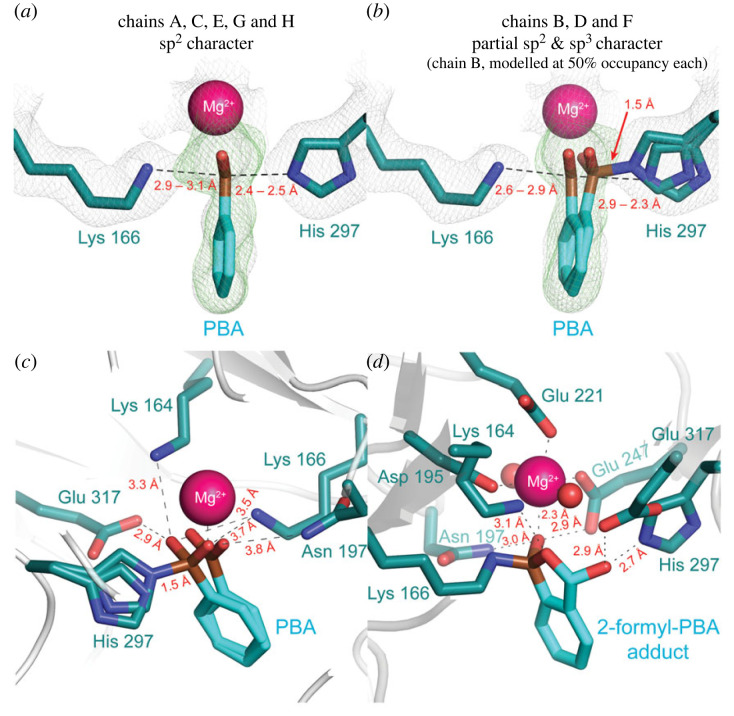
MR with bound boronic acid derivatives. MR-bound PBA (PDB ID: 6VIM [103]) was best modelled as having an sp^2^-hydridized boron in chains A, C, E, G and H of the MR homooctamer (panel (*a*)), but in chains B, D and F, PBA was best modelled by the species with an sp^2^-hydridized boron and the species with an sp^3^-hydridized boron forming an N*^ε^*^2^–B interaction with His 297, each at 50% occupancy (panel (*b*)). As shown in panel (*c*), the boronic acid hydroxyl groups of PBA form multiple H-bonds with the side chains of active site residues, in addition to the His 297 N*^ε^*^2^–B dative bond. Similarly, the potent inhibition exhibited by 2-formyl-PBA arises from formation of multiple H-bonds between the benzoxaborole adduct and the side chains of active site residues, as well as the Lys 166 N*^ζ^*–B dative bond (panel (*d*), PDB ID: 7MQX [105]). In all panels, the ligand and active site residues are shown in stick representation, and the Mg^2+^ is represented as a sphere. In panels (*a*,*b*), the 2F_o_ – F_c_ map is represented by a grey mesh contoured at 1.0*σ*, and the simulated annealing omit map (*F*_o_ – *F*_c_) is represented by a green mesh centred around PBA and contoured at 2.5*σ*. (Online version in colour.)

^11^B nuclear magnetic resonance (NMR) spectroscopy revealed an upfield shift of the ^11^B NMR signal from 28.2 ppm, corresponding to the sp^2^-hybridized boron atom of free PBA in solution at pH 7.5, to a value of 0.97 ppm, suggesting formation of an N–B interaction with the bound inhibitor existing either partially or fully in its anionic, sp^3^-hybridized state [103]. Interestingly, isothermal titration calorimetry experiments revealed that the binding affinity of PBA was reduced 2.8 × 10^3^-fold and 31-fold for the K166M, and H297N MR variants, respectively, relative to wild-type MR, indicating that Lys 166 contributed markedly to the binding affinity. Solution of the X-ray crystal structure of the MR·PBA complex to 2.00-Å resolution (PDB ID: 6VIM) revealed that the boron atom was located between the N*^ζ^* and N*^ε^*^2^ atoms of Lys 166 and His 297, respectively (figure 7*a,b*) [103]. Surprisingly, however, the side chain of Lys 166 formed an H-bond with the hydroxyl group of the boronic acid and did not participate in a N–B interaction. Instead, the electron density was consistent with the boron atom being sp^2^-hydridized (trigonal planar) in chains A, C, E, G and possibly H of the homooctameric structure, but in chains B, D and F, the boron could be modelled as both sp^2^-hydridized (trigonal) and as sp^3^-hydridized (tetrahedral), each at 50% occupancy, with the latter hydridization state arising from formation of a 1.5-Å N*^ε^*^2^–B dative bond with His 297. In addition to this N*^ε^*^2^–B interaction, the hydroxyl groups of the boronic acid formed H-bonds with the side chains of Lys 164, Lys 166, Asn 197, His 297 and Glu 317, as well as bidentate, electrostatic interactions with the Mg^2+^ ion at the active site (figure 7*c*). Thus, the remarkable binding affinity of PBA arises from multiple interactions between the boronic acid moiety and the catalytic machinery at the active site.

### (b) 2-Formylphenylboronic acid

*o*-Carbonyl arylboronic acids such as 2-formylphenylboronic acid (2-formyl-PBA) have been used to covalently modify the ε-NH_2_ group of Lys residues in proteins [102,106,107], as well as the N-terminus [108,109]. Since the ε-NH_2_ group of Lys 166 did not form an N–B interaction with the boron atom of PBA (*vide supra*), we anticipated that Lys 166 might form a Schiff base with the 2-formyl group that would subsequently be stabilized by direct coordination of the lone pair of electrons on the imine nitrogen to the boron atom to form an iminoboronate [107,110]. In accord with these expectations, we discovered that 2-formyl-PBA is a slow-onset inhibitor of MR, exhibiting *K*_i_ and *K*_i_* values of 5.1 µM and 0.26 µM, respectively [105], making it one of the most potent inhibitors of MR identified to-date that does not have any additional substituents on the phenyl ring (*K*_m_/*K*_i_* ≈ 3000, figure 3). Furthermore, substitution of Lys 166 by Arg obviated inhibition, confirming that the ε-NH_2_ group of Lys was essential for inhibition. In the absence of enzyme, ^11^B NMR spectroscopy revealed two signals for 2-formyl-PBA: one at 29.8 ppm corresponding to the neutral trigonal R–B(OH)_2_ group and the other at 8.5 ppm corresponding to the anionic tetrahedral R–B(OR)(OH)_2_^–^ group formed from the reversible cyclization to benzoxaborole [111]. Upon addition of MR, a new signal at 6.0 ppm was observed, which was the sole signal present when the enzyme was present in slight excess over the 2-formyl-PBA. Addition of a 12-fold excess of the competitive inhibitor BzH (5.0 mM), relative to the concentration of 2-formyl-PBA, displaced all the 2-formyl-PBA from the active site, regenerating the ^11^B NMR signals associated with free 2-formyl-PBA. These observations were consistent with reversible binding of the 2-formyl-PBA at the active site of MR and the observed ^11^B NMR chemical shift change suggested formation of a bound species possessing a negatively charged, tetrahedral boron atom. Counter to our expectations of iminoboronate formation and an interaction of the boronic acid moiety with MR that might resemble that of PBA, the X-ray crystal structure of MR with bound 2-formyl-PBA, solved to 1.91-Å resolution (PDB ID: 7MQX [105]), revealed that the ε-NH_2_ of Lys 166 formed a 1.5-Å N*^ζ^*–B dative bond with 2-formyl-PBA accompanied by cyclization to form a benzoxaborole adduct [111,112] rather than the expected iminoboronate (figure 7*d*). One hydroxyl group of the resulting anionic tetrahedral boronic acid adduct was coordinated to the Mg^2+^ ion (Mg^2+^–O distance of 2.3 Å), and the other hydroxyl group had reacted with the *o*-carbonyl group to form a cyclic hemiacetal. Despite these differences from the MR·PBA complex, the cyclic benzoxaborole adduct still formed H-bonding interactions (≤3.0 Å) with the side chains of key active site residues involved in catalysis, including Asn 197, Lys 164, His 297 and Glu 317, as well as the Mg^2+^-ligand Glu 247.

For both PBA and 2-formyl-PBA, van der Waals interactions with the phenyl ring ensure proper binding and orientation of these ligands at the active site so that the boronic acid moiety is positioned proximal to the enolization machinery of the active site. Potent inhibition then arises from the resulting boronic acid moieties clasping the adjacent key catalytic residues and the Mg^2+^ ion, in addition to the weak N*^ε^*^2^–B interaction between His 297 and PBA, or the N*^ζ^*–B bond formed between the ε-NH_2_ group of Lys 166 and 2-formyl-PBA.

## 7. Concluding remarks: capturing Δ*G*_tx_ for inhibitor binding

**Figure 8.**
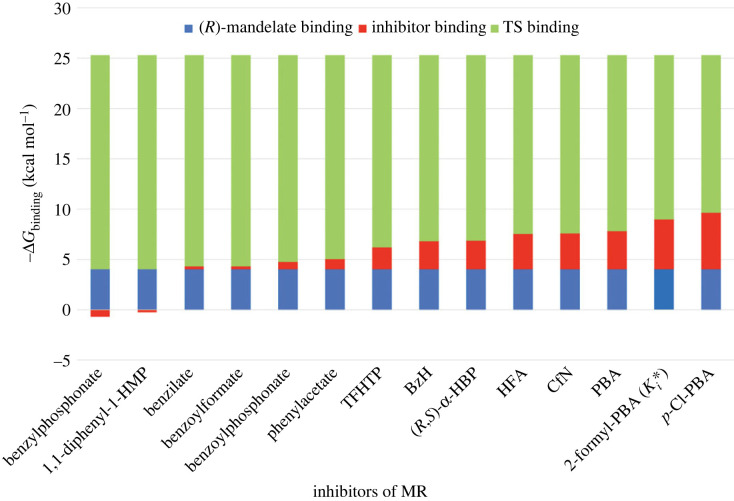
Free energy changes accompanying binding of the substrate (blue), various inhibitors (red) and the altered substrate in the TS (green) for MR. For benzylphosphonate and 1,1-diphenyl-1-HMP, the negative value corresponds to the free energy by which the binding free energy of the inhibitor is reduced relative to that of the substrate (*R*)-mandelate. (Online version in colour.)

Of the inhibitors developed for MR, ≤38% of the available Δ*G*_tx_ is captured by these inhibitors for binding (figure 8). Recognizing that Δ*G*_tx_ is a thermodynamic expression that encompasses *all* modes of catalysis [104], how can additional free energy of TS stabilization be captured to maximize inhibitor binding [113]? Development of high-affinity TS analogue inhibitors must capitalize on both the number and strength of the H-bonding, electrostatic interactions, and dispersion forces or van der Waals interactions, that accompany an enzyme's tightening grip on the altered substrate at the TS. For example, the failure of BzH, CfN and HFA to capture more than approximately 30% of the approximately 26 kcal mol^–1^ of the free energy of TS stabilization furnished by MR arises, in part, from the binding of these intermediate/TS analogues in the *Z*-conformation so that proton transfer from Glu 317 or H-bond formation are not possible. Moreover, these analogues are not dianionic, which diminishes their interaction with the Mg^2+^ ion relative to that expected for the *aci*-carboxylate intermediate. While the enhanced binding affinity of *p*-Cl-PBA appears to arise from the increased dispersion forces [11,113] between the *p*-Cl-phenyl moiety and the hydrophobic pocket, which occur at the TS, it is the ability of the boronic acid group in either PBA or the benzoxaborole adduct to engage in multiple interactions with critical active site residues and the Mg^2+^ ion that help capture more of the TS stabilization free energy. Furthermore, since optimum binding of the TS arises from cooperative interactions between the TS and the binding determinants [7,19,104], inhibitors that exhibit multiple interactions with the enzyme should be able to exploit these synergistic interactions. Thus, beyond simply using the surmised structural and electronic features of the TS to guide inhibitor design, considerations of maximizing interactions with multiple catalytic binding determinants may be integral to the design of effective TS analogue inhibitors capable of capturing a greater proportion of the free energy of TS stabilization for binding [114]. As Wolfenden pointed out, ‘The remaining difference may not be insurmountable … a few adjustments in structure might generate inhibitors of almost unlimited potency’ [104, p. 243].

## Data Availability

The data are provided in the electronic supplementary material [115].
